# Characteristics of blood immune cell profile and their correlation with disease progression in patients infected with HIV-1

**DOI:** 10.1186/s12879-023-08847-z

**Published:** 2023-12-20

**Authors:** Xiao-Yan Guo, Meng-Meng Qu, Xi Wang, Ze-Rui Wang, Jin-Wen Song, Bao-Peng Yang, Yun-Tian Guo, Yang Zhang, Chao Zhang, Xing Fan, Wen Xu, Ruonan Xu, Ji-Yuan Zhang, Si-Yuan Chen, Yan-Mei Jiao, Li-Jun Sun, Fu-Sheng Wang

**Affiliations:** 1 Senior Department of Infectious Diseases, The Fifth Medical Centre of Chinese PLA General Hospital, National Clinical Research Center for Infectious Diseases, Beijing, 100039 China; 2 Clinic of Center for Infection, Beijing Youan Hospital, Capital Medical University, Beijing, 100069 China; 3 Department of Gastroenterology, First Medical Center of Chinese PLA General Hospital, Beijing, 100853 China

**Keywords:** HIV-1, Mass cytometry, Myeloid cells, T cells, Disease progression

## Abstract

**Background:**

Antiretroviral therapy (ART) can reduce viral load in individuals infected with human immunodeficiency virus (HIV); however, some HIV-infected individuals still cannot achieve optimal immune recovery even after ART. Hence, we described the profile of peripheral immune cells and explored the association with disease progression in patients infected with HIV-1.

**Methods:**

Mass cytometry analysis was used to characterize the circulating immune cells of 20 treatment-naïve (TNs), 20 immunological non-responders (INRs), 20 immunological responders (IRs), and 10 healthy controls (HCs). Correlation analysis was conducted between cell subpopulation percentages and indicators including HIV-1 cell-associated (CA)-RNA, DNA, CD4^+^ T cell count, and CD4/CD8 ratio.

**Results:**

Global activation, immunosenescence, and exhaustion phenotypes were observed in myeloid cells and T cells from individuals with HIV-1 infection. We also found that specific subsets or clusters of myeloid, CD4^+^ T, and CD8^+^ T cells were significantly lost or increased in TN individuals, which could be partially restored after receiving ART. The percentages of several subpopulations correlated with HIV-1 CA-RNA, DNA, CD4^+^ T cell count, and CD4/CD8 ratio, suggesting that changes in immune cell composition were associated with therapeutic efficacy.

**Conclusion:**

These data provide a complete profile of immune cell subpopulations or clusters that are associated with disease progression during chronic HIV-1 infection, which will improve understanding regarding the mechanism of incomplete immune recovery in INRs.

**Supplementary Information:**

The online version contains supplementary material available at 10.1186/s12879-023-08847-z.

## Background

Human immunodeficiency virus-1 (HIV-1) infection remains a major global health burden as it causes considerable morbidity and mortality. Although antiretroviral therapy (ART) can significantly reduce viral load to undetectable levels in patients infected with HIV-1, approximately 10–40% of HIV-infected individuals who receive effective ART still cannot achieve optimal immune recovery [1, 2]. These patients are termed immunological non-responders (INRs) and exhibit poor prognosis and increased risks of mortality and morbidity compared to immunological responders (IRs) due to incomplete immune recovery [1, 3]. A CD4^+^ T cell count with a low nadir, long duration before ART initiation, coinfections, and later stages of disease progression are well-known factors associated with an incomplete immune recovery [4–7]. However, the alterations across all immune cell subsets between different treatment outcomes during chronic HIV-1 infection have not been completely identified. A deeper understanding of immune responses in HIV-1 infected patients is pivotal to depicting the characteristics of HIV infection and to reveal the mechanism of poor immune recovery in INRs.

Recently, high-dimensional mass cytometry (CyTOF) has been used to characterize the immune populations of individuals infected with HIV-1. By the CyTOF, several researches successfully uncovered novel immune cell subsets, such as specific CD4^+^ T cells and natural killing (NK) cells, or unique lymphocyte clusters [8–10]. It can also be used to describe myeloid and T-cell immune profiles during chronic HIV-1 infection. The CyTOF can overcome the dilemma generated by the great diversity of lymphocyte subpopulations and comprehensive characterization of immune cell subset alterations during chronic HIV-1 infection which the normal flow cytometry cannot handle.

Dendritic cells (DCs) and monocytes are innate immune cells of the myeloid lineage that play a crucial role in the early defenses against viruses as a bridge between innate and adaptive immunity [11–13]. In HIV or Simian immunodeficiency virus infections, classical DCs (cDCs) have impaired antigen presentation and cytokine production, resulting in inefficient T-cell proliferation [14, 15]. Moreover, dysregulation of cDCs is also identified as an indicator of disease progression in early infection [16, 17]. Several studies have indicated that plasmacytoid DCs (pDCs) exhibit dysregulated immunophenotypic attributes in patients exhibiting HIV-1^+^ viremia [18]. The frequency of pDCs is reduced in primary patients infected with HIV-1, and the cell count is restored to CD4 counts in early ART [19]. Monocytes are dysregulated during HIV infection and their turnover is linked to disease progression [20]. Furthermore, specific monocyte and DC subpopulations are found in the early stages of HIV infection [12]. A more activated phenotype of monocytes and DCs is associated with poor CD4^+^ T cell recovery in INRs [21]. However, few studies have discussed the detailed subset differences in myeloid cells between INRs and IRs.

HIV-1 infection is characterized by continued HIV-1 replication, decline in circulating CD4^+^ T lymphocytes, and over-activation and exhaustion of CD8^+^ T lymphocytes [22–24]. It is well documented that loss of naïve CD4^+^ T cells, naïve CD8^+^ T cells, and memory CD4^+^ T cells is accompanied by an increase in memory and effector CD8^+^ T cells in individuals infected with HIV-1 [22, 25]. In addition, HIV-1 persistence results in activation and progressive exhaustion of T cells. For example, exhausted cytotoxic CD8^+^ T cells lose their effector function to eradicate virus-infected cells and differentiate between effector cells and memory cells [26]. T cell exhaustion is believed to be a major obstacle in the incomplete recovery of CD4^+^ T cells. INRs have higher percentages of exhausted T cells and show a more activated and differentiated stage than IRs [23, 27]. Numerous studies have focused on newly identified T cell subsets and their dysfunctional mechanisms [28, 29]. However, studies simultaneously assessing myeloid and T cell compositions and and their correlation with disease progression are still lacking.

To better understand the immune profiles during chronic HIV-1 infection, we carried out mass cytometry analysis to unravel the alterations in immune cell subpopulations from individuals across the four groups (TNs, IRs, INRs and HCs). Meanwhile, we also comprehensively analyzed the immunological characteristics and alterations in immune cell subsets or clusters responsible for better or poor prognosis in patients undergoing ART. Overall, this study describes the global immune cell profile of myeloid and T cells and provides clues for a deeper understanding of the immune mechanism of poor immune recovery in INRs.

## Materials and methods

### Study participants

In this study, we enrolled a cohort of 10 HCs, 20 TN individuals with chronic HIV-1 infection prepared to receive ART, and 40 individuals who had undergone ART for > 2 years, with no reported ART interruptions and undetectable plasma HIV-1 RNA after at least two consecutive tests. Patients with incomplete clinical information, drug resistance, or co-infection with other viruses were excluded. Based on the CD4^+^ T cell counts at the time of collection, there were 20 IRs (CD4^+^ T cell counts ≥ 500 cells/µL) and 20 INRs (CD4^+^ T cell counts ≤ 350 cells/µL).

### Quantification of HIV DNA and RNA

Total DNA and RNA were separately extracted from thawed peripheral blood mononuclear cells (PBMCs) samples using the QIAamp DNA Mini Kit (Qiagen, Valencia, California, USA) and HiPure Total RNA Plus Mini Kit (Magen, Guangzhou, China), respectively. HIV-1 DNA and cell-associated HIV-1 RNA (CA-RNA) values were calculated per million total PBMCs using quantitative PCR.

### Mass cytometry staining and data acquisition

The total 43 metal-conjugated antibodies used in this study and their corresponding providers and clones are listed in Table S1. Metal-labeled antibodies were purchased or prepared using the MaxPAR Antibody Labelling Kit (Fluidigm) according to the manufacturer’s instructions. Conjugated antibodies were titrated to determine their optimal concentration before use.

Frozen PBMCs were thawed in complete media and counted with retained more than 85% viability, after which 1 million PBMCs from each participant were washed with 1× PBS and stained with Cisplatin to exclude dead cells. Fc receptor blocking solution was added, and then the cells were stained with cell surface markers for 30 min at 4 °C, washed twice with fluorescence-activated cell sorting (FACS) buffer (1× PBS + 0.5% bovine serum albumin), and fixed in DNA intercalator-191/193Ir solution (Maxpar Fix and Perm Buffer, Fluidigm) overnight to discriminate single-nucleated cells from doublets, followed by washing and staining with intracellular antibody mix for 30 min at 4 °C. Finally, the cells were washed and resuspended in deionized water with 20% EQ beads (Fluidigm) and were ready for running on a mass cytometer (Helios, Fluidigm).

### CyTOF data analysis

Data analysis was performed using theflow cytometry standard (FCS) concatenation tool from Cytobank, debarcoded using a doublet filtering scheme with mass-tagged barcodes, and manually gated using the FlowJo V10 software to exclude debris, dead cells and doublets, thus retaining live, singlet, and valid immune cells. To visualize high-dimensional data, the t-distributed stochastic neighbor embedding (t-SNE) algorithm was performed on all samples and among each group. To visualize the expression analyses on t-SNE maps, expression was normalized between 0 and 1. Heatmaps were displayed in R using a complex heatmap, and expression was also normalized between 0 and 1.

### Statistical analysis

Data analysis was performed using GraphPad Prism 8.0 (GraphPad Software, San Diego, CA, USA). The results were compared using the Mann–Whitney U test t, Kruskal-Wallis test, Chi-Square test and continuity correction. Spearman’s correlation coefficient was used to assess the correlation between the two variables.

## Results

### General characteristics of the study populations

**Fig. 1:**
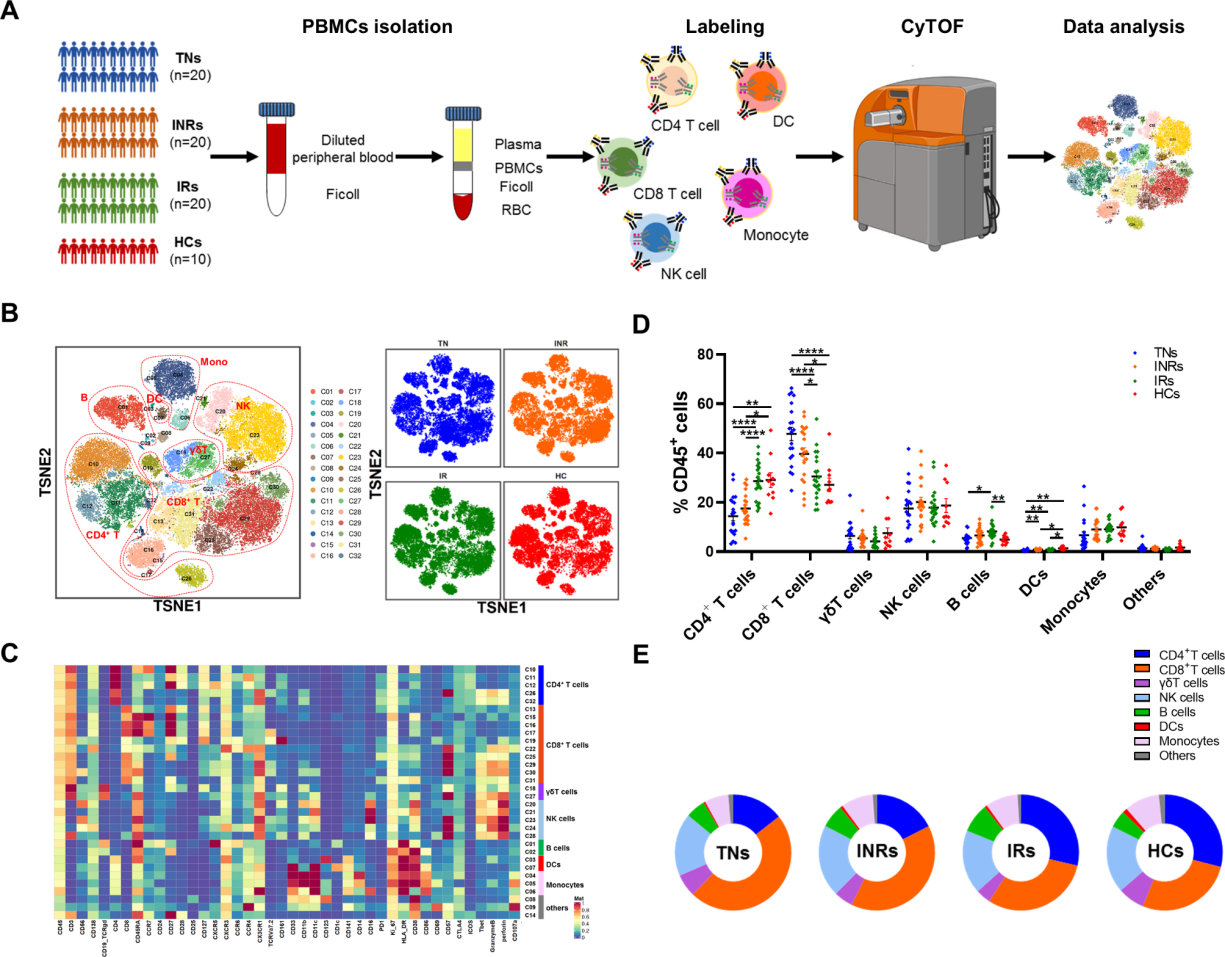
Study design and characterization of major immune cell subsets from PBMCs. **(A)** Overview of workflow. PBMCs were isolated from healthy individuals (n = 10) and individuals infected with HIV-1 including TN individuals (n = 20), INRs (n = 20) and IRs (n = 20). PBMCs were stained using metal-labeled antibodies to simultaneously quantitate the levels of 43 different surface and intracellular proteins followed by acquisition using CyTOF2 (Fluidigm) and analysis using t-Distributed Stochastic Neighbor Embedding (t-SNE). **(B)** T-SNE projection of PBMCs showing major cell clusters based on expression of cell type-specific makers. Each dot corresponds to a single cell and colored according to PhenoGraph clustering (left); groups are each colored as indicated (right). **(C)** Heatmap showing the median metal intensity of individual markers for each cluster as indicated. **(D)** Frequency of major immune cell subsets including CD4^+^ T cells, CD8^+^ T cells, γδT cells, NK cells, B cells, DCs, and monocytes from individuals with chronic HIV-1 infection and healthy individuals. Groups are shown in different colors. Horizontal lines represent mean values, and each dot represents one sample. Significant differences are indicated by *P < 0.05; **P < 0.01; ***P < 0.001; ****P < 0.0001. Differences between each group were analyzed using a two-sided unpaired Mann–Whitney U-test. **(E)** Donut chart showing the composition of CD45^+^ cells colored according to major cell types from each cohort. TNs: treatment-naïve HIV-1-infected individuals; INRs: immune non-responders to antiretroviral therapy; IRs: immune responders to antiretroviral therapy; HCs: healthy controls

We recruited 70 individuals classified as TNs (n = 20), INRs (n = 20), IRs (n = 20) and HCs (n = 10) (Fig. 1A). Detailed clinical information on participant demographics, including age, CD4^+^ T cell count, CD8^+^ T cell count and viral load, is shown in Table 1. All individuals were men, with a median age of 30–45 years. The INRs experienced poor immune recovery and had a significantly lower CD4^+^ T cell count of 295 (IQR, 282–327) cells/µL than IRs [976 (IQR, 719–1,148) cells/µL, *P* < 0.001]. INR participants had a median CD8^+^ T cell count of 808/µL compared to 1059/µL in IRs, and had a lower CD4/CD8 ratio (0.43 vs. 1.06, *P* < 0.001) than IRs. There were no differences in the years of ART or components of the ART regimen between INRs and IRs.

**Table 1:** Demographic characteristics of enrolled participants in this study

	TNs	INRs	IRs	HCs	*P*_value	
Age (years)	38 (30–45)	38 (34–43)	38(32–42)	38(31–43)	0.910^a^	
CD4^+ ^T (cells/µL)	348 (154–468)	295 (282–327)	976 (719–1148)	820 (517–1080)	0.000^a^	
CD8^+ ^T (cells/µL)	1021 (649–1136)	808 (495–1014)	1059 (760–1346)	806 (705–953)	0.227^a^	
CD4/CD8 ratio	0.41 (0.15–0.50)	0.43 (0.30–0.51)	1.06 (0.65–1.40)	1.07(0.76–1.46)	0.000^a^	
Viral load (log_10_ copies/mL)	4.21 (3.76–4.67)	<LDL	<LDL	-	-	
ART time (years)		6.1 (4–8)	6.6 (5-8.3)	-	0.531^b^	
ART regimen (%)						
AZT/3TC/EFV	-	2 (10%)	6 (30%)	-	0.236^c^	
TDF/3TC/EFV	-	14 (70%)	13 (65%)	-	1.000^d^	
TDF/3TC/DTG		2 (10%)	0		0.468^c^	
TAF/FTC/DTG	-	2 (10%)	1 (5%)	-	1.000^c^	

All indicators except gender and ART regimen are shown as median(interquartile range)

TNs: treatment-naïve HIV-1-infected individuals; INRs: immune non-responders to antiretroviral therapy; IRs: immune responders to antiretroviral therapy; HCs: healthy controls; ART: antiretroviral therapy; AZT: azidothymidine; 3TC: lamivudine; EFV: efavirenz; TDF: tenofovirdisoproxil; DTG: dolutegravir; TAF: tenofovir alafenamide; FTC: emtricitabine; LDL: lower than detectable level

^a^Kruskal-Wallis test; ^b^Mann-Whitney test; ^c^continuity correction; ^d^Chi-Square test

### Major immune cell subset compositions in individuals with chronic HIV-1 infection

A 43-parameter CyTOF panel was established to simultaneously phenotype immune cells for markers of differentiation and activation states, co-stimulatory/co-inhibitory molecules, cytokines and chemokine receptors (Table S1).

Seven major cell subsets were identified, including CD4^+^ T cells (CD3^+^CD4^+^), CD8^+^ T cells (CD3^+^CD8^+^), γδ T cells (CD3^+^TCRγδ^+^), NK cells (CD3^−^CD56^+^), B cells (CD3^−^CD19^+^), DCs (CD123^+^ and CD11c^+^), and monocytes (CD33^+^CD14^+^ or CD16^+^) (Fig. 1B, C). To investigate the distinctive immune profiles during chronic HIV-1 infection, we analyzed the distribution of each subset across three groups of individuals infected with HIV-1 and HCs (Fig. 1D, E). Overall, HIV-1-infected individuals exhibited an increase in CD8^+^ T cells and a reduction in CD4^+^ T cells, DCs, and monocytes, especially in the TN group. In addition, the percentage of CD8^+^ T cells decreased, whereas the percentages of CD4^+^ T cells, DCs, and monocytes increased after receiving ART. A higher percentage of CD8^+^ T cells and a lower percentage of CD4^+^ T cells were also observed in INRs than in IRs. Thus, we provide a global description of the immune cell composition in the peripheral blood of chronic HIV-1-infected individuals and HCs.

### Alterations in innate myeloid cell composition and their correlation with viral reservoir and immune recovery

**Fig. 2:**
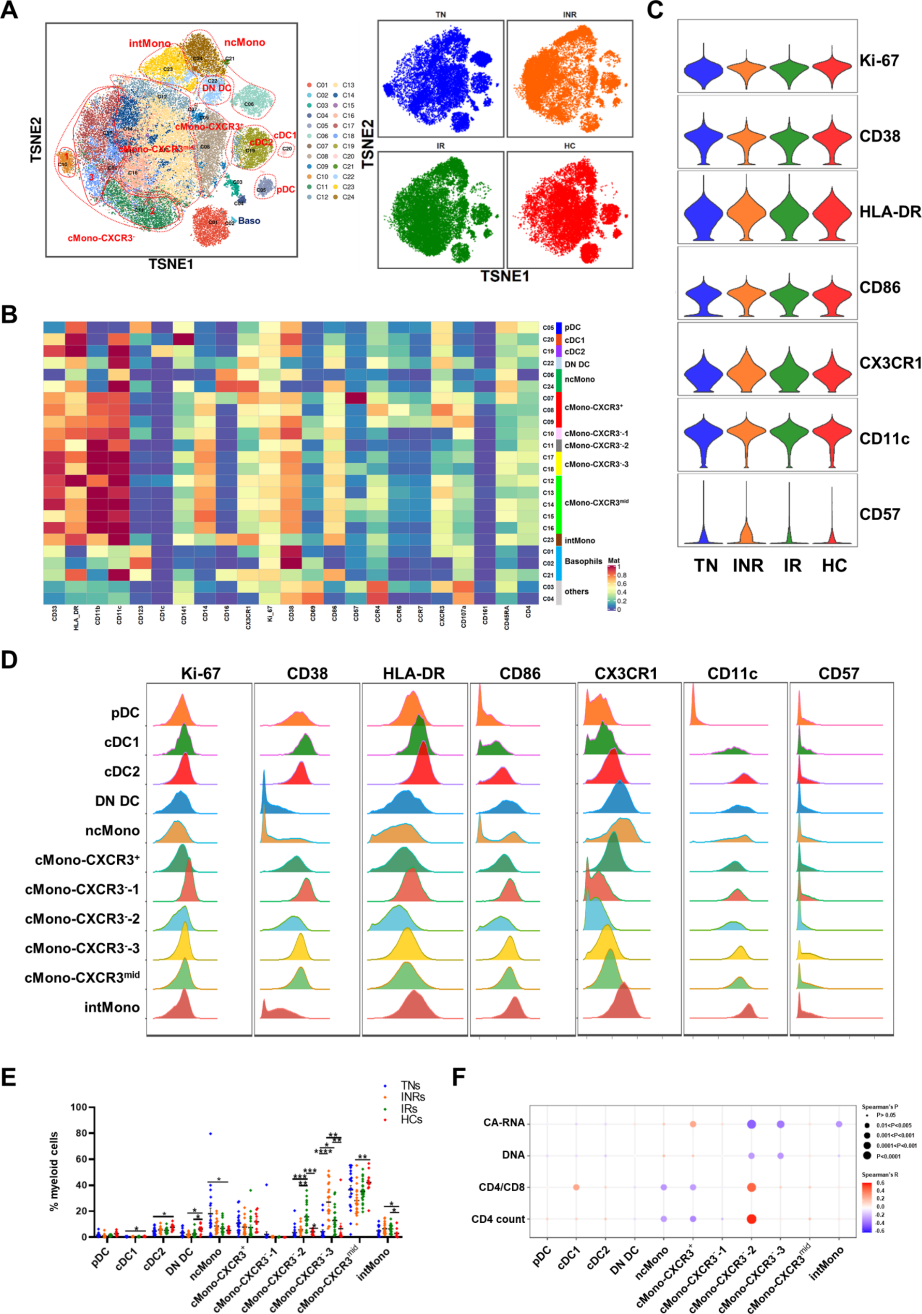
Characterization of myeloid cells from PBMCs of TNs, INRs, IRs and HCs. **(A)** A viSNE map of gated myeloid live single cells depicting the myeloid cell landscape. Different groups are colored as indicated (right). **(B)** Heatmap showing the median metal intensity of individual markers for each cluster as indicated. **(C)** Violin plots showing the expression of selected markers on global myeloid cells of HCs and patients with chronic HIV-1 infection. **(D)** Histograms showing expression of indicated markers for 11 myeloid cell subpopulations, including pDC, cDC1, cDC2, DN DC, non-classical monocytes (ncMono), detailed classical monocytes (cMono) according to CXCR3 expression, and intermediate monocytes (intMono). **(E)** Dot plot showing the frequencies of myeloid cell subsets from individuals with chronic HIV-1 infection and healthy individuals. Groups are shown in different colors. Horizontal lines represent mean values, and each dot represents one sample. Significant differences are indicated by *P < 0.05; **P < 0.01; ***P < 0.001. Differences between each group were analyzed using a two-sided unpaired Mann–Whitney U-test. **(F)** Bubble heatmap showing Spearman’s rank correlation of viral reservoir parameters, CD4/CD8 ratio, and CD4^+^ T cell count with myeloid cell subset percentages. The color of the bubble represents the correlation coefficient, the deeper the more relevant between the two variables, while the direction is indicated by colors: red for a positive correlation and blue for a negative correlation. The size of the bubble indicates statistically significant *P-values*, with larger values representing greater significance

To address the distribution of detailed cell subsets and clusters across different chronic HIV-1-infected groups and HCs, we chose myeloid and T cells for in-depth analysis. Dimensional reduction analysis t-SNE was applied to the marker expression of myeloid cell data, and 13 cell types or subtypes were identified according to the expression of canonical markers (Fig. 2A, B). The subsets were pDC (CD123^+^HLA-DR^+^), cDC1 (CD11c^+^HLA-DR^+^CD141^+^), cDC2 (CD11c^+^HLA-DR^+^CD1c^+^), double-negative DC (DN DC, CD11c^+^HLA-DR^+^CD1c^−^CD141^−^), non-classical monocytes (ncMono, CD33^+^CD14^+^CD16^++^), classical monocytes (cMono, CD33^+^CD14^++^CD16^−^), intermediate monocytes (intMono, CD33^+^CD14^++^CD16^+^), basophils (CD123^+^HLA-DR^−^) and other mixed cells that could not be easily identified were termed as others and excluded from further analysis. Five cMono subpopulations were further classified as cMono-CXRC3^+^, cMono-CXCR3^−^-1,2,3, and cMono-CXCR3^mid^ according to the expression of CXCR3, CX3CR1, and CD107a. We also compared the activation (CD38, HLA-DR, CD86), immunosenescence (CD57) and cell proliferate (Ki-67) states of whole myeloid cells among different groups. Compared to HCs, the expression of CD38, HLA-DR, CD86, and CD57 was slightly higher in individuals infected with HIV-1 (Fig. 2C). Moreover, the INR group showed a more activated and immunosenescence state of myeloid cells compared to the IR group.

We then evaluated the distribution of each subset across the four groups (TNs, INRs, IRs, and HCs). As shown in Fig. 2E, the percentages of cDC1, cDC2, and DN DC decreased in individuals with chronic HIV-1 infection, and the percentages of cDC1 and cDC2 increased after receiving ART but did not return to normal levels as detected in the HCs. The proportions of ncMono and intMono in individuals with chronic HIV-1 infection were higher than HCs. CX3CR1 showed robust expression in these two subsets, indicating individuals infected with HIV-1 were manifesting an inflammatory state (Fig. 2D). Of particular interest, the percentage of the intMono subset further increased after ART. These cells were more highly activated than the others, characterized by higher expression of HLA-DR and CD86 (Fig. 2D). In contrast, the proportions of cMono-CXCR3^−^-2 and cMono-CXCR3^−^-3 showed the opposite response in individuals with chronic HIV-1 infection after ART. There was a lower percentage of cMono-CXCR3^−^-2, but a higher percentage of cMono-CXCR3^−^-3 subset in INRs than IRs (Fig. 2E). As shown in Fig. 2D, the cMono-CXCR3^−^-3 subset exhibited higher expression of Ki-67, CD38, HLA-DR, CD86, CD11c, as well as CD57, which is a hallmark indicator of immunosenescence for T and NK cells, suggesting that enrichment of cMono-CXCR3^−^-3 may contribute to poor immune recovery in INRs. Altogether, these data revealed the myeloid cell composition under the four conditions during chronic HIV-1 infection.

We next assessed whether HIV-1 DNA and CA-RNA correlated with subset percentages in individuals with chronic HIV-1 infection (Fig. 2F, Table S2). There was a positive correlation between the cMono-CXCR3^+^ percentage of the general myeloid subset and HIV-1 CA-RNA (*r* = 0.2887, *P* = 0.028). We also found significant negative correlations between the percentage of cMono-CXCR3^−^-2 in general myeloid cells with HIV-1 CA-RNA (*r* = -0.4378, *P* = 0.0006) and HIV-1 DNA (*r* = -0.2819, *P* = 0.0321). Similarly, the correlation between HIV-1 CA-RNA (*r* = -0.3740, *P* = 0.0038) and HIV-1 DNA (*r* = -0.2920, *P* = 0.0262) was negative for the percentage of cMono-CXCR3^−^-3 subsets.

We investigated whether the fraction of cells within each subset was correlated with CD4^+^ T cell count and the CD4/CD8 ratio. As shown in Fig. 2F, the percentage of cDC1 in myeloid cells was positively correlated with the CD4/CD8 ratio. Both ncMono and cMono-CXCR3^+^ population percentages were negatively correlated with CD4^+^ T cell count and CD4/CD8 ratio (Table S2). Conversely, the percentage of cMono-CXCR3^−^-2 was significantly positively correlated with the CD4^+^ T cell count and CD4/CD8 ratio (Table S2). Taken together, our analysis demonstrated that these myeloid cell subpopulations, which are highly associated with the CD4^+^ T cell count, CD4/CD8 ratio, and viral reservoir, might be related to poor immune recovery in INRs.

### Distinct CD4^+^ T cell subsets and their correlation with HIV-1 disease-related parameters

**Fig. 3:**
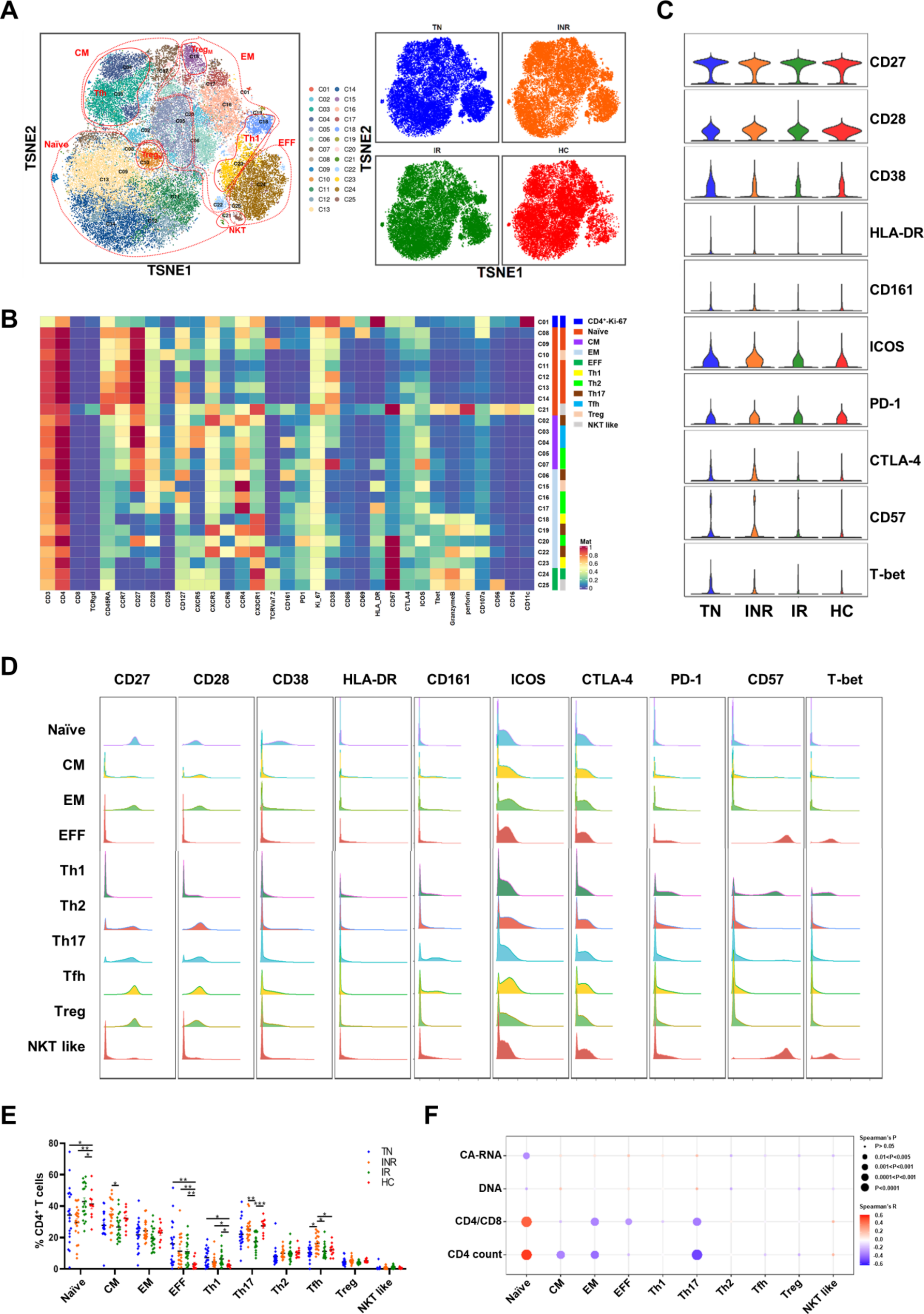
Characterization of CD4^+^ T cell subsets in individuals with chronic HIV-1 infection and HCs. **(A)** t-SNE map displaying CD4^+^ T cell clusters based on manual annotation of PhenoGraph clustering. Clusters are circled according to major cell subpopulations (left), and different groups are each colored as indicated (right). **(B)** Heatmap showing normalized expression of the markers from panel for PhenoGraph clustering. **(C)** Violin plots showing the expression distribution of selected cell markers of CD4^+^ T cells among the four groups. **(D)** Histograms showing expression of indicated markers for major CD4^+^ T cell subpopulations. **(E)** Dot plot showing the proportions of major CD4^+^ T cell subsets across the four groups. Different groups are shown with the same color as in** A. C.** Significant differences were indicated by *P < 0.05; **P < 0.01; ***P < 0.001. Differences between each group were analyzed using a two-sided unpaired Mann–Whitney U-test. **(F)** Bubble heatmap showing Spearman’s rank correlation of CD4^+^ T cell subset proportions with viral reservoir parameters, CD4/CD8 ratio, and CD4^+^ T cell count. The color of the bubble represents the correlation coefficient, the deeper the more relevant between the two variables, while the direction is indicated by colors: red for a positive correlation and blue for a negative correlation. The size of the bubble indicates statistically significant *P-values*, with larger values representing greater significance

The hallmark of HIV-1 infection is the progressive loss of CD4^+^ T cells together with activation and functional impairment [25]. According to the expression of canonical markers independent of individuals’ origins, the major CD4^+^ T cell subpopulations were Proliferating (Ki-67^++^, C01), Naïve (CD45RA^+^CCR7^+^CD27^+^), Central Memory (CM, CD45RA^−^CCR7^+^CD27^+^), Effector Memory (EM, CD45RA^−^CCR7^−^CD27^−^), and Effector (EFF, CD45RA^+^CCR7^−^CD27^−^) (Fig. 3A, B). Additionally, classic type 1 helper T (Th1), classic type 2 helper T (Th2) and type 17 helper T (Th17) cells were characterized by the expression of three makers as indicated: Th1 (CCR6^−^CCR4^−^CXCR3^+^), Th2 (CCR6^−^CCR4^+^) and Th17 (CCR6^+^CCR4^+^). Two separated regulatory T cell clusters (Treg, CD25^+^CD127^low^CXCR5^−^, C10 and C15) were identified. Notably, a subpopulation of follicular helper T cells expressing CXCR5, PD-1, and ICOS was distinguished into two clusters (C03: CD161^−^; C04: CD161^+^). Another subpopulation was termed natural killer T (NKT)-like cells with high expression of CD56, perforin, and granzyme B (GZMB). The expression of immune activation markers (CD27, CD28, CD38, HLA-DR), stimulating ligand (CD161), stimulating molecule (ICOS), inhibitory molecules (PD-1, CTLA-4), immunosenescence (CD57), and transcription factor (T-bet) in PBMCs from the four groups were visualized using a violin plot (Fig. 3C). We found that CD4^+^ T cell activation, immunosenescence and exhaustion were significantly higher in TN individuals, and decreased but not to levels as HCs after ART. The expression of the co-stimulatory molecules ICOS and CTLA-4 was higher in INRs than in IRs and HCs. In the CD4^+^ T cell subpopulation, EFF and NKT like cells were highly expressed in CD57 and T-bet, while other subpopulations were highly expressed in CD27 and CD28 (Fig. 3D).

**Fig. 4:**
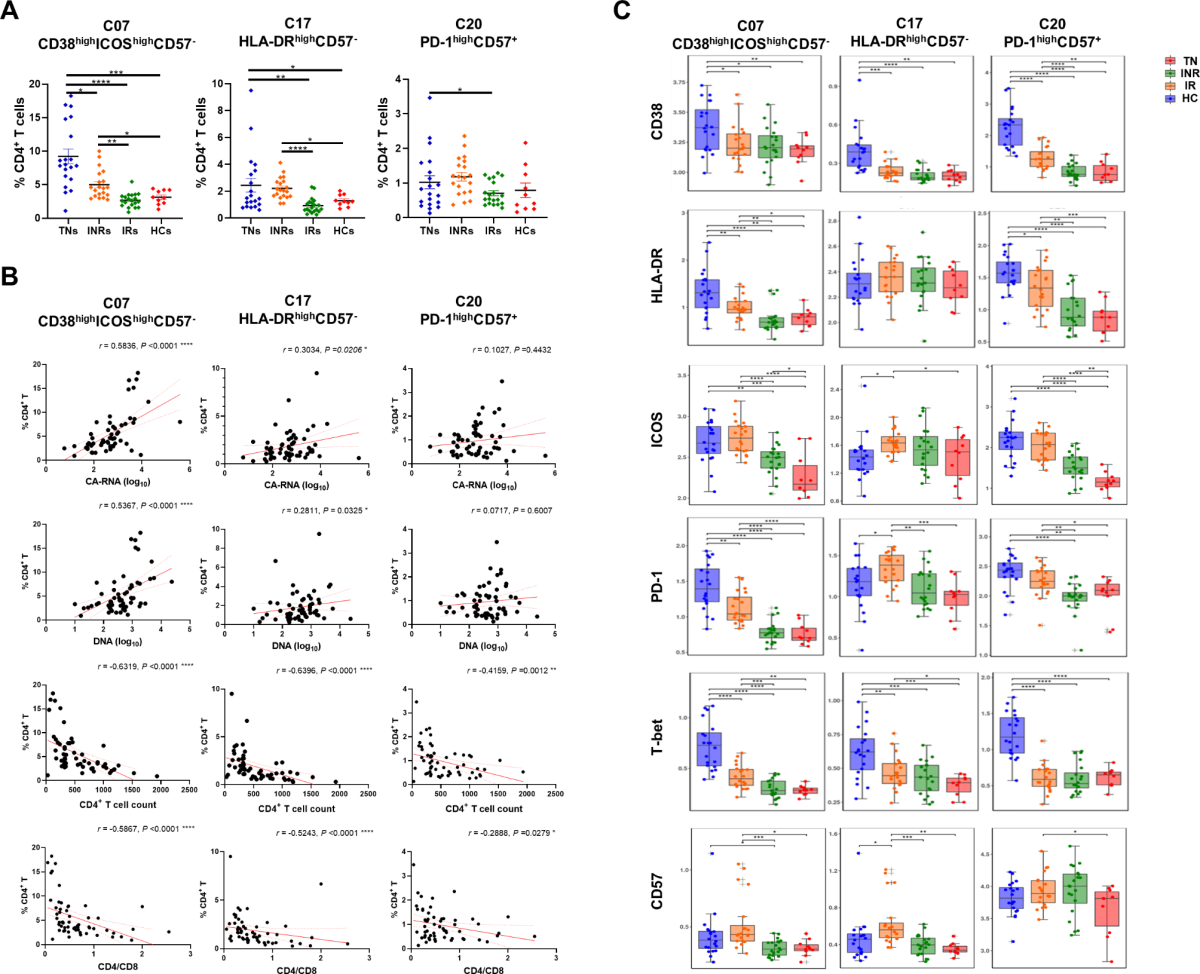
In-depth characterization of the CD4^+^ T cell compartment. **(A)** Percentage analyses of Cluster 07 (CD38^high^ICOS^high^CD57^−^), Cluster 17 (HLA-DR^high^CD57^−^) and Cluster 20 (PD-1^high^CD57^+^) of CD4^+^ T cells among conditions. Significant differences are indicated by *P < 0.05; **P < 0.01; ***P < 0.001; ****P < 0.0001. **(B)** Spearman’s rank correlation plot of the CD4/CD8 ratio, CD4^+^ T count, and viral reservoir parameters with proportions of the three clusters. Each dot represents one participant. **(C)** Boxplots showing the expression of selected markers for the above mentioned three CD4^+^ T clusters. Groups are shown in different colors. Horizontal lines represent median values, with whiskers extending to the farthest data point within a maximum of the 1.5× interquartile range. *P < 0.01; **P < 0.001; ***P < 0.0001. Differences between each group were analyzed using a two-sided unpaired Mann–Whitney U-test

We then investigated the distribution of CD4^+^ T cell subsets in the various cohorts. There were reduced proportions of naïve cells and increased proportions of EFF cells, which were not restored to the levels detected in HCs after receiving ART (Fig. 3E). The proportion of Th1 cells was higher, whereas the frequencies of Th2 and Th17 cells were lower in TN individuals (Fig. 3E). The frequencies of Th17 and follicular helper T cell (Tfh) cells were higher than those of IRs and HCs. Interestingly, in the deep analysis, we also compared the differences in each cluster and found that in INRs, the frequencies of cluster C07 (CD38^high^ICOS^high^CD57^−^), C17 (HLA-DR^high^CD57^−^) and C20 (PD-1^high^CD57^+^) were elevated compared with those in IRs and HCs (Fig. 4A, C).

We further evaluated the correlation between the frequency of CD4^+^ T cell subsets or clusters, viral reservoirs, and clinical indicators. The frequency of CD4-naïve cells was negatively correlated with HIV-1 CA-RNA (*r* = -0.3169, *P* = 0.0153) and HIV-1 DNA (*r* = -0.2360, *P* = 0.0745) (Fig. 3F, Table S3). A significant positive association was detected between the frequency of naïve cells and CD4^+^ T cell count (*r* = 0.5828, *P* < 0.0001) and the CD4/CD8 ratio (*r* = 0.4944, *P* < 0.0001) (Fig. 3F). The frequencies of CM, EM, EFF, and Th2 cells were negatively correlated with HIV-1 CA-RNA and DNA levels (Fig. 3F). As shown in Fig. 4B, the HIV-1 viral reservoir was positive with the frequencies of cluster C07, C17 and C20. In addition, the frequencies of these three clusters were significant negatively correlated with the CD4^+^ T cell count and CD4/CD8 ratio (Table S3). Thus, we depicted the composition of CD4^+^ T cell subsets and clusters, and their association with viral reservoirs and clinical factors in the peripheral blood of individuals with chronic HIV-1 infection.

### Changes of CD8^+^ T cell subsets and clusters and their association with disease progression

**Fig. 5:**
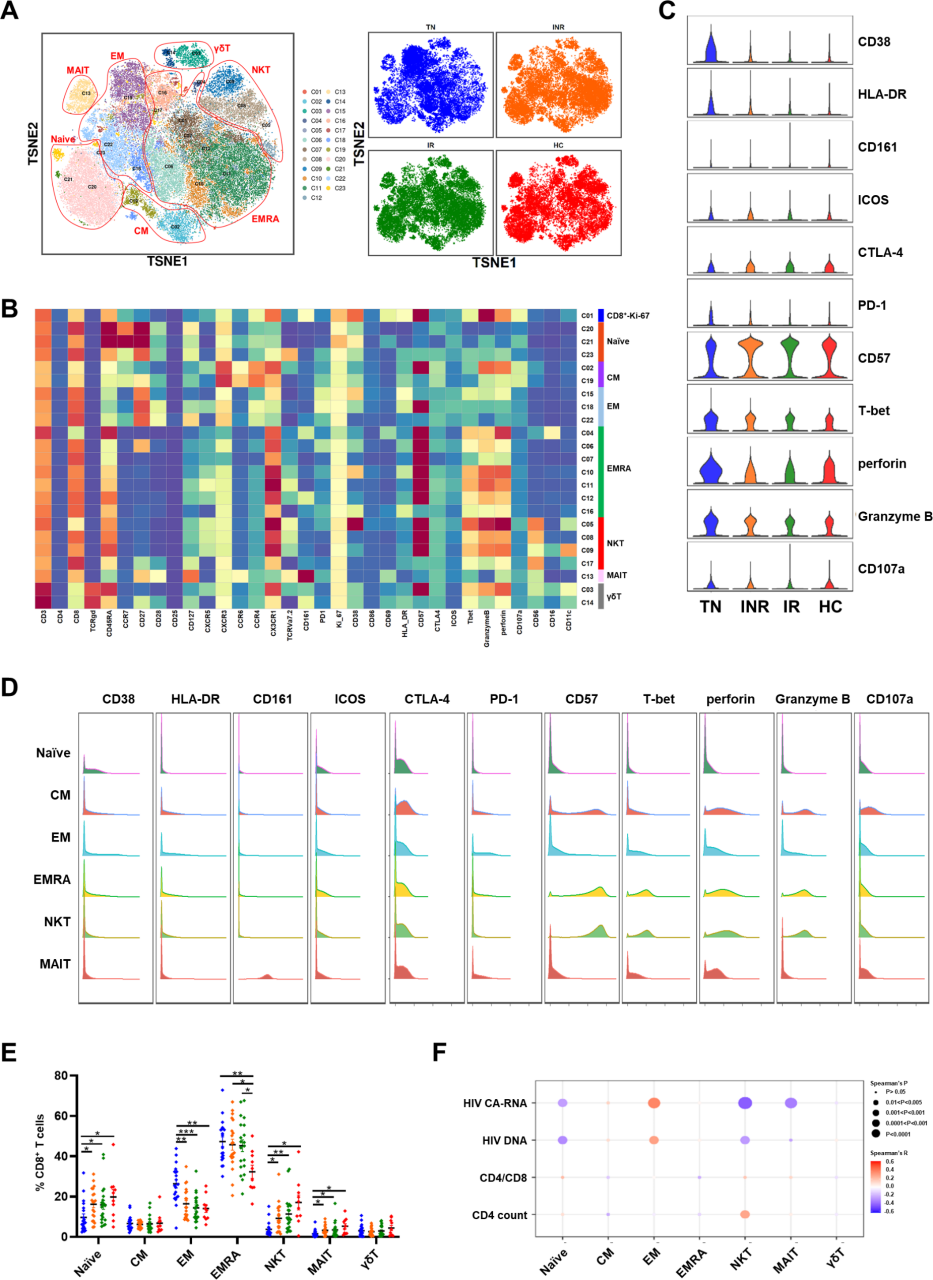
Characterization of CD8^+^ T cell subsets from PBMCs from individuals with HIV-1 infection and HCs. **(A)** t-SNE map showing CD8^+^ T cells from the PhenoGraph cluster identified in Fig. 5B, colored by cluster. Major CD8^+^ T cell subsets (left) and different groups (right) are each colored as indicated. **(B)** Heatmap showing normalized expression of the panel markers identified with PhenoGraph. Clusters were manually assigned to the CD8^+^ T cell subpopulations as indicated. **(C)** Violin plots displaying the expression of indicated cell markers for global CD8^+^ T cells across the four groups. **(D)** Histograms showing expression of selected markers for major CD8^+^ T cell subsets. **(E)** Dot plot showing six major CD8^+^ T cell subset percentages from the four group individuals. Different cohorts are shown with the same color in **A. C.** Significant differences are indicated by *P < 0.05; **P < 0.01; ***P < 0.001. Differences between each group were analyzed using a two-sided unpaired Mann–Whitney U-test. **(F)** Bubble heatmap showing Spearman’s correlation of CD8^+^ T cell subset percentages with viral reservoir parameters, CD4/CD8 ratio, and CD4^+^ T count. The color of the bubble represents the correlation coefficient, the deeper the more relevant between the two variables, while the direction is indicated by colors: red for a positive correlation and blue for a negative correlation. The size of the bubble indicates statistically significant P-values, with larger values representing greater significance

CD8^+^ T cell exhaustion, characterized by phenotypic and functional CD8^+^ T cell abnormalities, was found in HIV-1 infected individuals despite years of ART. Here, we defined CD8^+^ T cell subpopulations according to the expression of canonical markers. The major subsets of CD8^+^ T cells were Proliferating (Ki-67^++^, C01), Naïve (CD45RA^+^CCR7^+^CD27^+^), CM (CD45RA^−^CCR7^+^CD27^+^), EM (CD45RA^−^CCR7^−^CD27^−^), terminal effector memory T (EMRA, CD45RA^+^CCR7^−^CD27^−^), NKT (CD3^+^CD56^+^), mucosal-associated invariant T (MAIT, TCR Vα7.2^+^CD161^++^) and γδT (TCRγδ^+^) cells (Fig. 5A, B). Moreover, global CD8 + T cells in TN individuals showed higher activation and cytotoxicity than patients undergoing ART and HCs (Fig. 5C). INRs displayed higher expression of activation markers (CD38 and HLA-DR), co-stimulatory molecules (ICOS and CTLA-4), exhaustion and immunosenescence compared to IRs and HCs. The CD8 + T cell subpopulations CM, EMRA and NKT were highly expressed in CD57 and higher cytotoxicity (Fig. 5D).

We assessed CD8^+^ T cell subset composition among individuals with and without HIV-1 infection. The proportion of naïve cells decreased, while the percentages of EM and EMRA cells increased in TN individuals, INRs, and IRs compared to that in HCs (Fig. 5E). The proportions peaked in TN individuals, but did not return to the levels detected in HCs. The frequencies of the NKT and MAIT cytotoxicity subsets were lower in individuals infected with HIV-1. The frequencies of NKT and MAIT cells were lower in INRs than in IRs or HCs. Correlation analysis showed a significant negative correlation between the HIV-1 reservoir and the percentage of CD8^+^-naïve, NKT, and MAIT cells (Fig. 5F, Table S4). However, the analysis showed highly positive associations between the percentage of CD8^+^ EM cells and HIV-1 DNA and CA-RNA, indicating that these subpopulations may predict disease prognosis for individuals infected with HIV-1 after receiving ART (Table S4).

**Fig. 6:**
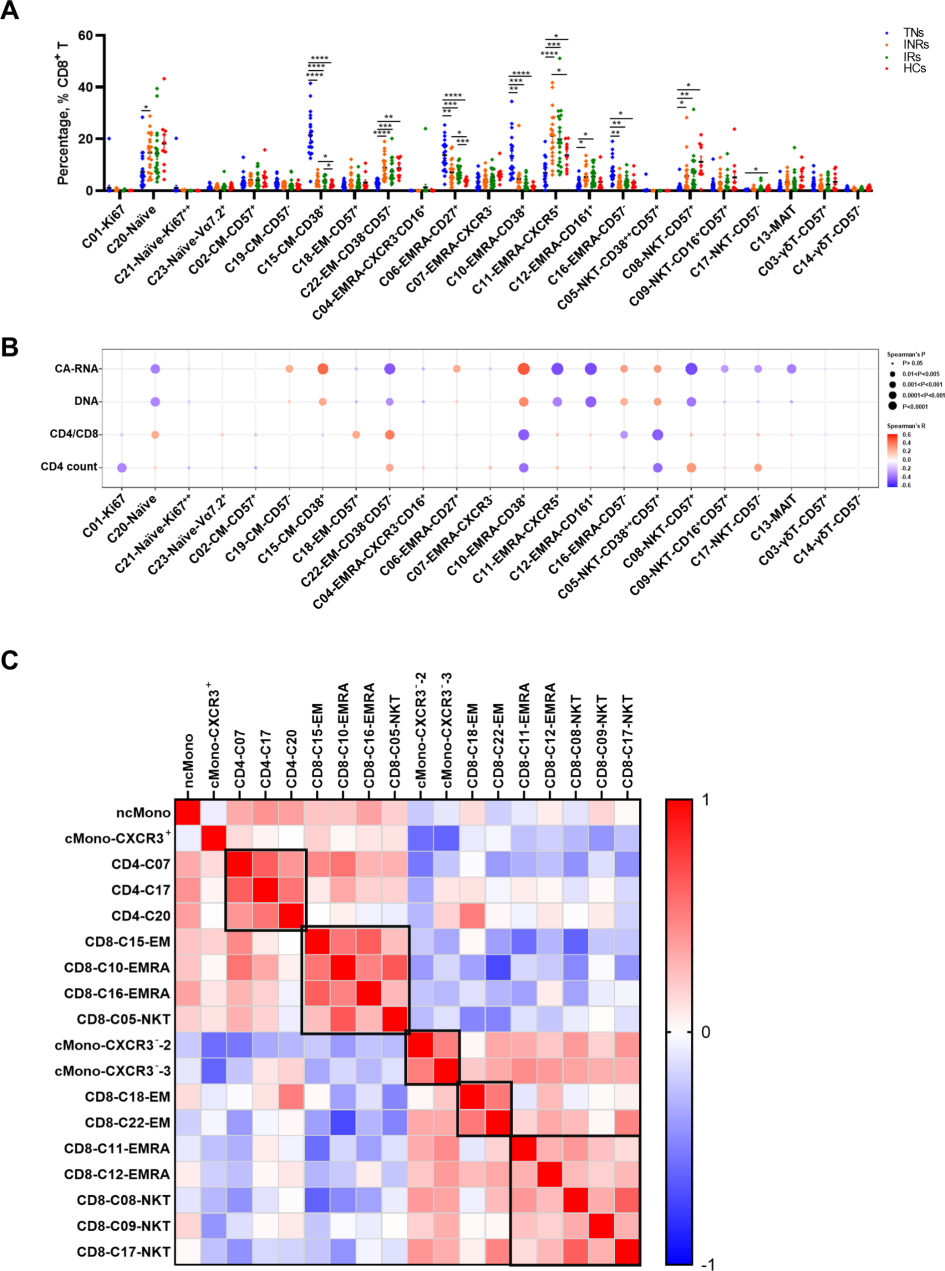
In-depth characterization of the CD8^+^ T cell clusters and relationship between proportions of various subpopulations. **(A)** Dot plot showing the percentages of CD8^+^ T cell clusters across the four conditions. Significant differences are indicated by *P < 0.05; **P < 0.01; ***P < 0.001; ****P < 0.0001. Differences between each group were analyzed using a two-sided unpaired Mann–Whitney U-test. **(B)** Bubble heatmap showing Spearman’s correlation of CD8^+^ T cell cluster percentages with viral reservoir parameters, CD4/CD8 ratio, and CD4^+^ T cell count. The color of the bubble represents the correlation coefficient, the deeper the more relevant between the two variables, while red and blue indicate a positive and negative correlation. respectively. The size of the bubble indicates statistically significant P-values, with larger values representing greater significance. **(C)** Heatmap showing Spearman correlations between the frequencies of selected cell subsets and clusters as indicated. Red and blue indicate positive and negative associations, respectively. The deeper the more relevant between the two variables.

To further examine the cell cluster composition and its correlation with the CD4^+^ T cell count, CD4/CD8 ratio, and viral reservoir, we compared the percentages of CD8^+^ T cell clusters according to the order indicated in the heatmap (Figs. 5B and 6A). Two EM clusters were identified in our data: C15 with high expression of CD38, which comprised a large percentage of cells from TN individuals and decreased after ART (Fig. 6A); Another C18, highly expressing CD57, was not enriched in TN individuals, and the percentage was negatively associated with HIV-1 CA-RNA and DNA. The proportion of C18 significantly correlated with the CD4/CD8 ratio (Fig. 6B). The frequencies of C06-EMRA-CD27^+^, C10-EMRA-CD38^+^, C16-EMRA-CD161^+^ and C05-NKT-CD38^++^/CD57^+^ were higher in the TN group and reduced after ART, but did not return to normal levels as in HCs. Association results showed that the proportions of CD8^+^ T cells were associated with worse clinical outcomes (Fig. 6B). Furthermore, patients undergoing ART had higher levels of C11-EMRA-CXCR5^+^, C12-EMRA-CD161^+^, and three NKT clusters than did treatment-naïve individuals. Correlation analysis indicated that these clusters were closely associated with better clinical recovery.

### Correlation analysis of cell subsets and clusters that significantly related to disease outcomes

To systematically quantify the relationships between immune cell subsets and clusters present in HIV-1 infection, we chose immune cell subsets or clusters in which the frequency was significantly associated with viral reservoir and immune recovery and used their frequencies for further correlation analyses. Multiple robust relationships were also identified. The frequency of ncMono cells was high in samples with high levels of clusters C07, C17, and C20 in CD4^+^ T cells, which also contained high amounts of C15-EM, C10-EMRA, C16-EMRA, and C05-NKT CD8^+^ T cells (Fig. 6C). We found that the proportion of these cells was higher in the INRs than in the IRs and HCs. Collectively, the correlation analysis indicated that the enrichment of these subpopulations may represent poor immune recovery in individuals with chronic HIV-1 infection.

In contrast, both cMono-CXCR3^−^-2 and cMono-CXCR3^−^-3 percentages in myeloid cells were negatively correlated with the viral reservoir and frequency of ncMono cells, showing the opposite response during HIV-1 infection. Lower percentages of C11-EMRA and C12-EMRA were found in individuals with low levels of the three NKT clusters and MAIT cells, which are characterized by high expression of cytotoxicity markers, such as GZMB and perforin. These functional clusters had lower percentages in INRs than in IRs and HCs, except for C11-EMRA. In addition, significant positive associations were detected between the frequencies of these subsets, suggesting that decreased frequencies might be associated with incomplete immune recovery in individuals infected with HIV-1.

## Discussion

Myeloid and T cells play important roles in the immune response against HIV-1. Previous studies have clarified some immune cell characteristics, particularly those of T cells in chronic HIV-1 infected patients [8, 25, 30]. Moreover, DCs and monocytes participate in inflammatory events and regulate adaptive immunity, thereby affecting viral persistence [12, 21]. However, an integrated analysis to characterize the dynamics of myeloid and T cell subsets and their correlation with disease-related parameters that eventually reflect disease outcomes is still lacking. In this study, we report a comprehensive profile of immune cells among TNs, INRs, IRs, and HCs using mass cytometry, which not only illustrates the circulating innate and adaptive immune cell component variations but also identifies specific cell subsets and clusters that correlate closely with disease progression outcomes.

Noticeably, we found that compared to HCs, the frequency of ncMono and intMono was higher in HIV-1-infected patients. ncMono and intMono secrete higher levels of pro-inflammatory cytokines and chemokines upon stimulation, in contrast to their cMono counterparts [31, 32]. The ncMono percentage in myeloid cells was higher in INRs than in IRs, indicating a more pro-inflammatory environment that might correlate with a higher incidence of comorbidities in INR patients. In addition, we found a global loss of DC subsets in HIV-1-infected individuals and the frequency of cDC1 and cDC2 increased after treatment. This change may be due to the rapid migration of peripheral DCs to the lymph nodes after HIV-1 infection to initiate adaptive immunity [33]. Although CD4^+^ T cells are typically recognized as the main target of HIV infection, other cell types such as circulating DCs and monocytes can also harbor integrated, replication-competent HIV [34, 35]. A complete HIV cure cannot be achieved without the elimination of all viral reservoirs. Therefore, these potential myeloid reservoirs of persistent HIV-1 infection require further characterization and should be considered when designing HIV-1 cure strategies. A significant loss of naive CD4^+^ and CD8^+^ T cell subsets and expansion of the EMRA CD8^+^ T cell subset with high cytotoxic capacity was observed in TNs, which were partially restored upon treatment. These results are consistent with those of our previous report [30].

Tregs downregulate immune activation through cell-to-cell contact or secretion of cytokines to exert anti-inflammatory effects and participate in the pathogenesis of HIV-1 infection [36]. Here, we also observed higher percentages of Tregs in INRs than in IRs. Among the CD4^+^ T cell clusters characterized in our study, three clusters were significantly enriched in INRs compared to IRs and HCs. Interestingly, C 07 expresses high levels of the activation marker CD38 and co-stimulatory molecule ICOS, C 17 expresses high levels of the activation markers HLA-DR, and C 20 exhibits a high expression of the immunosenescent marker CD57 and exhaustion indicator PD-1, suggesting that these clusters play a vital role in persistent overactivation, immunosenescence, and exhaustion. Within CD8^+^ T cells, NKT and MAIT cells were lost in individuals with TN and increased after receiving ART. However, the change in the proportion of MAIT cells between INRs and IRs was not as obvious as in other reports [37, 38]. This phenomenon may have occurred because the CD4^+^ T cell count used to define INRs and IRs varies among studies. The median CD4^+^ T cell count was 295 cells/µL in the INRs in this study, which is dissimilar to that in other studies.

We also investigated the variation in myeloid and T cell subpopulations among different conditions and determined their association with the peripheral CD4^+^ T cell count and CD4/CD8 ratio to evaluate host immune recovery. Here, we observed that ncMono and cMono-CXRC3^+^, which expressed high levels of CD38, CXCR3, and CX3CR1, were higher in TNs, lower in INRs and IRs, and negatively correlated with the CD4^+^ T cell count and CD4/CD8 ratio. The persistent aberrant activation of monocytes likely contributes to the incomplete recovery of T-cell effector functions in INRs. Conversely, we discovered a lower percentage of a subpopulation, cMono-CXCR3^−^-2, which might be an indicator of better immune recovery.

We also investigated the variation in myeloid and T cell subpopulations among different conditions and determined their association with the peripheral CD4^+^ T cell count and CD4/CD8 ratio to evaluate host immune recovery. Here, we observed that ncMono and cMono-CXRC3^+^, which expressed high levels of CD38, CXCR3, and CX3CR1, were higher in TN individuals, lower in INRs and IRs, and negatively correlated with the CD4^+^ T cell count and CD4/CD8 ratio. The persistent aberrant activation of monocytes likely contributes to the incomplete recovery of T-cell effector functions in INRs. Conversely, we discovered a lower percentage of a subpopulation, termed cMono-CXCR3^−^-2, compared with immunological responders, which was positively associated with the CD4^+^ T cell count and CD4/CD8 ratio, suggesting that the presence of cMono-CXCR3^−^-2 may be an indicator of better immune recovery.

Together with the higher proportions of INRs than IRs, the frequencies of the three CD4^+^ T cell clusters mentioned above were significantly negatively correlated with the CD4^+^ T cell count and CD4/CD8 ratio. Within CD8^+^ T cells, C10-EMRA-CD38^+^ cells that exhibited high levels of GZMB and perforin expression were abundant in TN individuals and decreased after ART, but not to the same levels as HCs. Correlation analysis suggested that higher percentages of these CD4^+^ and CD8^+^ T cell clusters indicate poor clinical outcomes despite ART. Functional studies are necessary to better characterize the role of these clusters in chronic HIV-1 infection. It has been reported that the baseline CD4^+^ cell count, CD4/CD8 ratio, naïve CD4^+^ T cell count, and one-year CD4^+^ T cell count after ART initiation were better predictors for immune recovery prognosis under long-term therapy [12, 39]. These indicators were not analyzed in this study and will be considered in future research.

Nc Mono, cMono-CXCR3^+^, CD4-C07, CD4-C17, CD4-C20, C15-EM, C10-EMRA, C16-EMRA, and C05-NKT CD8^+^ T cells were positively correlated HIV-1 viral reservoirs. These cells had higher activation, and these cells may have a reciprocal promotion effect. In addition, cMono-CXCR3^−^-2, cMono-CXCR3^−^-3, C11-EMRA-CXCR5^+^, C12-EMRA-CD161^+^, C18-EM-CD57^+^, C22-EM-CD38^−^CD57^−^ CD8^+^ T cells and C08-NKT-CD57^+^, C09-NKT-CD16^+^CD57^+^, and C17-NKT-CD57^−^ have a positive correlation with low activation levels and may have an important role in checking and balancing the highly activated cells. This also provides direction for balancing the ratio and interrelationship of these cells to restore CD4^+^ T cell level to halt disease progression.

HIV-1 DNA and CA-RNA are both important indicators to estimate the viral reservoir size. Here, we analyzed their associations with immune cell subpopulation percentages among the TNs, INRs, and IRs. Similarly, we found that both DNA and CA-RNA levels from peripheral blood were higher in INRs than in IRs, as indicated in our previous report [40]. The results of this study showed that the frequency of cMono-CXCR3^−^-2 was positively associated with the CD4 T cell count and CD4/CD8 ratio but inversely correlated with HIV-1 CA-RNA and DNA, suggesting that the presence of cMono-CXCR3^−^-2 may be a candidate indicator for predicting disease outcome. Meanwhile, cMono-CXRC3^+^ with high expression of CD38, CXCR3, and CX3CR1 was positively associated with viral reservoirs, in line with a previous report [12]. The percentage of NKT and MAIT cells was negatively associated with HIV-1 CA-RNA and DNA. Moreover, the majority of NKT cell clusters were negatively correlated with the viral reservoirs. Our data revealed that the HIV-1 reservoir was positively associated with C06-EMRA-CD27^+^ and C10-EMRA-CD38^+^ cells, which displayed high expression of CD27 and CD38, respectively. Thus, the underlying molecular mechanisms of these clusters require further elucidation.

Our study has several limitations. First, due to the limited number of parameters for mass cytometry and the aim to evaluate the comprehensive immune landscape, we were unable to include more receptors and functional markers in this analysis. Future studies, including additional markers, will help elucidate the dynamics of cell subpopulations. Second, only peripheral blood samples were obtained in this study, and lymphoid tissues, such as lymph nodes and gut-associated lymphoid tissue, which are major sites of HIV persistence, are needed to further analyze the paired immune cell composition with our phenotyping data.

## Conclusion

Taken together, our results reveal the diversity and various immune cell compositions among TNs, INRs, IRs, and HCs and their correlation with HIV-1 reservoirs and clinical parameters. Several cell subsets and clusters with significant associations were identified, indicating their important roles in chronic HIV-1 infection. In particular, we found that cMono-CXCR3^−^-2 may be a better indicator of immune recovery. These results contribute to a better understanding of the mechanisms driving incomplete immune recovery in INRs.

### Electronic supplementary material

Below is the link to the electronic supplementary material.

Supplementary Material 1

## Data Availability

The datasets used during the current study are available from the corresponding author on reasonable request.

## Abbreviations

ART: Antiretroviral therapy
CA-RNA: cell-associated RNA
cDCs: classical DCs
CM: Central Memory
cMono: classical monocytes
CyTOF: high-dimensional mass cytometry
DCs: Dendritic cells
DN DC: double-negative DC
EM: Effector Memory
EMRA: Terminal effector memory T
FACS: fluorescence-activated cell sorting
FCS: Fluorescence Confocal Spectroscopy
GZMB: granzyme B
HCs: healthy controls
HIV-1: Human immunodeficiency virus-1
INRs: immune non-responders
IRs: immune responders
intMono: intermediate monocytes
MAIT: mucosal-associated invariant T
ncMono: non-classical monocytes
NK: natural killing
NKT: natural killer T
PBMCs: peripheral blood mononuclear cells
pDCs: plasmacytoid DCs
Tfh: follicular helper T cell
Th1: type 1 helper T
Th2: type 2 helper T
Th17: type 17 helper T
TNs: treatment-naïve HIV-1-infected individuals
t-SNE: t-stochastic neighbor embedding
