# Supplementary material for: Characteristics of blood immune cell profile and their correlation with disease progression in patients infected with HIV-1
Source: BMC Infect Dis. 2023 Dec 20;23:893. doi: 10.1186/s12879-023-08847-z (PMC10731693; doi:10.1186/s12879-023-08847-z)
Supplement: Supplementary file 1 — Supplementary Material 1 [file 12879_2023_8847_MOESM1_ESM.docx]

Supplementary Tables

Table S1. List of CyTOF antibodies used in this study.

| No. | Antigen Target | Clone | Elemental Isotope | Purpose |
| --- | --- | --- | --- | --- |
| 1 | CD45 | HI30 | 89Y | Leukocytes |
| 2 | CD3 | UCHT1 | 115In | Pan T cell, NKT-Like cells |
| 3 | Ki-67 | SolA15 | 139La | Proliferation |
| 4 | CD56 | NCAM16.2 | 141Pr | Pan NK cell, γδT cell activation |
| 5 | TCR γ/δ | 5A6.E9 | 142Nd | Pan γδ T cell |
| 6 | CD19 | HIB19 | 142Nd | B cells |
| 7 | CD196(CCR6) | G034E3 | 143Nd | Chemokine receptor; T cell and B cell differentiation |
| 8 | CD28 | CD28.2 | 144Nd | T cell and NK cell differentiation |
| 9 | CD69 | FN50 | 145Nd | Early marker of lymphocyte activation |
| 10 | CD123(IL-3Rα) | 6H6 | 146Nd | Plasmacytoid dendritic cells |
| 11 | CCR7(CCR7) | G043H7 | 147Sm | T cell differentiation |
| 12 | CD1c | L161 | 148Nd | Dendritic cells, NKT-Like cells |
| 13 | CD25 | 24212 | 149Sm | Regulatory T cells |
| 14 | CD14 | M5E2 | 150Nd | Monocyte differentiation |
| 15 | CD107a | H4A3 | 151Eu | Cytotoxic T cells, NK cells |
| 16 | CD27 | O323 | 152Sm | T and B cell differentiation |
| 17 | CD161 | HP-3G10 | 153Eu | T cell subsets, NK cells |
| 18 | CD24 | ML5 | 154Sm | B cell differentiation |
| 19 | CD45RA | HI100 | 155Gd | T cell and dendritic cell differentiation |
| 20 | CD33 | WM53 | 156Gd | Myeloid progenitors, mono, gran, DC |
| 21 | CD183(CXCR3) | G025H7 | 157Gd | Dendritic cell, T cell, and B cell differentiation |
| 22 | CD194(CCR4) | L291H4 | 158Gd | Th1 and Th17 cells |
| 23 | CD11c | BU15 | 159Tb | Dendritic Cell differentiation |
| 24 | CX3CR1 | K0124E1 | 160Gd | Chemokine receptor |
| 25 | CD152(CTLA-4) | 14D3 | 161Dy | Activated T and B cells |
| 26 | CD278(ICOS) | C398.4A | 162Dy | Activated T cells |
| 27 | CD138 | DL101 | 163Dy | Plasma cells, pre-B cells |
| 28 | CD141 | M80 | 164Dy | Dendritic cell differentiation |
| 29 | T-bet* | 4B10 | 165Ho | Th1 cells, T cell development/differentiation |
| 30 | TCRVα7.2 | 3C10 | 166Er | Mait cells |
| 31 | Granzyme B* | QA16A02 | 167Er | Cytotoxic T cells, NK cells |
| 32 | CD57 | HNK-1 | 168Er | NK and CD8+ T cell immune senescence |
| 33 | CD185(CXCR5) | RF8B2 | 169Tm | Mature B cells, T follicular helper cells |
| 34 | CD127(IL-7Rα) | A019D5 | 170Er | Cytokine receptor; T cell differentiation |
| 35 | CD86 | Fun-1 | 171Yb | Mono, activated B and T cells |
| 36 | CD38 | HIT2 | 172Yb | Monocyte, dendritic cell, T cell, and B cell activation/differentiation |
| 37 | perforin* | B-D48 | 173Yb | NK cells and CTL (CD8+) |
| 38 | CD279(PD-1) | EH12.2H7 | 174Yb | T cell inhibitory receptor |
| 39 | CD16 | 3G8 | 175Lu | Monocyte, NK cell, and dendritic cell differentiation |
| 40 | HLA-DR | L243 | 176Yb | T cell and monocyte activation, NK cell lineage |
| 41 | CD4 | RPA-T4 | 197Au | CD4 T and NKT-Like cells |
| 42 | CD8 | RPA-T8 | 198Pt | CD8 T, NK, and NKT-Like cells |
| 43 | CD11b | M1/70 | 209Bi | Myeloid cells, NK cells |

*Intracellular antibodies

Table S2. Spearman’s rank correlation of viral reservoir parameters, CD4/CD8 ratio and CD4 count with the percentage of myeloid cell subsets.

|  | pDC | cDC1 | cDC2 | DN DC | ncMono | cMono-CXCR3^+^ | cMono-CXCR3^-^-1 | cMono-CXCR3^-^-2 | cMono-CXCR3^-^-3 | cMono-CXCR3^mid^ | intMono |
| --- | --- | --- | --- | --- | --- | --- | --- | --- | --- | --- | --- |
| CA-RNA |  |  |  |  |  |  |  |  |  |  |  |
| *r* | -0.03144 | -0.1099 | 0.1096 | 0.08467 | 0.1904 | 0.2887 | 0.1148 | -0.4378 | -0.374 | 0.09848 | -0.2965 |
| *P* | 0.8148 | 0.4114 | 0.4126 | 0.5275 | 0.1522 | 0.0280 | 0.3909 | 0.0006 | 0.0038 | 0.4621 | 0.0238 |
| CA-DNA |  |  |  |  |  |  |  |  |  |  |  |
| *r* | -0.1079 | -0.1544 | -0.09571 | 0.003046 | 0.2299 | 0.1756 | -0.01588 | -0.374 | -0.292 | 0.07783 | -0.1529 |
| *P* | 0.4200 | 0.2473 | 0.4748 | 0.9819 | 0.0825 | 0.1873 | 0.9058 | 0.0321 | 0.0262 | 0.5614 | 0.2519 |
| CD4/CD8 |  |  |  |  |  |  |  |  |  |  |  |
| *r* | 0.0319 | 0.2918 | 0.1472 | -0.08854 | -0.2867 | -0.2693 | -0.04095 | 0.4834 | 0.1587 | 0.1626 | 0.07036 |
| *P* | 0.8121 | 0.0262 | 0.2700 | 0.5086 | 0.0291 | 0.0409 | 0.7602 | 0.0001 | 0.2341 | 0.2227 | 0.5997 |
| CD4 |  |  |  |  |  |  |  |  |  |  |  |
| *r* | 0.1509 | 0.1505 | 0.1923 | -0.09162 | -0.2717 | -0.3344 | -0.188 | 0.6108 | 0.07276 | 0.2101 | 0.0834 |
| *P* | 0.2581 | 0.2595 | 0.1482 | 0.4940 | 0.0391 | 0.0103 | 0.1576 | <0.0001 | 0.5873 | 0.1134 | 0.5337 |

Table S3. Spearman’s rank correlation of viral reservoir parameters, CD4/CD8 ratio and CD4 count with the percentage of CD4^+^ T cell subpopulations.

|  | Naïve | CM | EM | EFF | Th1 | Th2 | Th17 | Tfh | Treg | NKT like |
| --- | --- | --- | --- | --- | --- | --- | --- | --- | --- | --- |
| CA-RNA |  |  |  |  |  |  |  |  |  |  |
| *r* | -0.3169 | 0.09876 | 0.09405 | 0.1868 | 0.09759 | 0.2131 | -0.2182 | -0.1085 | -0.1654 | -0.1913 |
| *P* | 0.0153 | 0.4608 | 0.4825 | 0.1602 | 0.4661 | 0.1082 | 0.0998 | 0.4174 | 0.2147 | 0.1502 |
| CA-DNA |  |  |  |  |  |  |  |  |  |  |
| *r* | -0.236 | 0.1918 | -0.02818 | 0.1115 | 0.01818 | 0.2544 | -0.2383 | -0.02098 | -0.1882 | -0.1213 |
| *P* | 0.0745 | 0.1492 | 0.8337 | 0.4046 | 0.8922 | 0.0539 | 0.0717 | 0.8758 | 0.157 | 0.3644 |
| CD4/CD8 |  |  |  |  |  |  |  |  |  |  |
| *r* | 0.4944 | -0.1871 | -0.3404 | -0.2909 | -0.1504 | -0.3615 | 0.02892 | -0.02609 | -0.01997 | 0.2367 |
| *P* | <0.0001 | 0.1596 | 0.0089 | 0.0268 | 0.2596 | 0.0053 | 0.8294 | 0.8459 | 0.8817 | 0.0736 |
| CD4 |  |  |  |  |  |  |  |  |  |  |
| *r* | 0.5828 | -0.344 | -0.4037 | -0.1864 | -0.01108 | -0.5144 | 0.00523 | -0.1615 | -0.223 | 0.2417 |
| *P* | <0.0001 | 0.0082 | 0.0017 | 0.1611 | 0.9342 | <0.0001 | 0.9689 | 0.226 | 0.0924 | 0.0676 |

Table S4. Spearman’s rank correlation of viral reservoir parameters, CD4/CD8 ratio and CD4 count with the percentage of CD8^+^ T cell subpopulations.

|  | Naïve | CM | EM | EMRA | NKT | MAIT | γδT |
| --- | --- | --- | --- | --- | --- | --- | --- |
| CA-RNA |  |  |  |  |  |  |  |
| *r* | -0.2664 | 0.1456 | 0.3925 | 0.02904 | -0.4475 | -0.3556 | -0.07805 |
| *P* | 0.0432 | 0.2753 | 0.0023 | 0.8287 | 0.0004 | 0.0062 | 0.5603 |
| CA-DNA |  |  |  |  |  |  |  |
| *r* | -0.3205 | 0.1151 | 0.3103 | 0.04565 | -0.2797 | -0.2571 | 0.05051 |
| *P* | 0.0142 | 0.3894 | 0.0178 | 0.7336 | 0.0335 | 0.0514 | 0.7065 |
| CD4/CD8 |  |  |  |  |  |  |  |
| *r* | 0.2219 | -0.1505 | 0.02212 | -0.2297 | 0.1856 | 0.1337 | -0.08867 |
| *P* | 0.0941 | 0.2593 | 0.8691 | 0.0829 | 0.163 | 0.3169 | 0.5081 |
| CD4 |  |  |  |  |  |  |  |
| *r* | 0.1059 | -0.232 | -0.04981 | -0.1142 | 0.2929 | 0.05593 | -0.05965 |
| *P* | 0.429 | 0.0797 | 0.7104 | 0.3933 | 0.0257 | 0.6767 | 0.6565 |
